# Keep on Keepin’ On: Investigating ACES and Positivity among Bereaved Black Middle Aged and Older Adults

**DOI:** 10.1093/geroni/igab046.3156

**Published:** 2021-12-17

**Authors:** Danielle McDuffie

**Affiliations:** The University of Alabama, Tuscaloosa, Alabama, United States

## Abstract

Many of the most damaging life events are more prevalent among Black older adults. Black people have been found to have higher amounts of adverse childhood experiences (ACEs), which are linked to detrimental life impacts. Additionally, bereavement occurs at a higher rate among Black people and older adults. Despite these challenges, Black older adults have been repeatedly cited overcoming these challenges. Accordingly, the present study sought to investigate whether Black middle to older aged adults who encountered two of life’s most difficult challenges (i.e. bereavement and ACEs) would still maintain positivity. 103 middle to older Black adults (M=44.72, SD=5.48, 67% male) from a larger online grief study were probed about factors including time since loss, positive outlook, and ACES. A linear regression and mediation analysis were used to analyze the data. ACES were found to significantly predict positive outlook among bereaved middle to older Black adults (F=11.46, p=.001), such that as the number of ACES increased, so did positivity in spite of bereavement. Notably, this association was not mediated by time since loss. Results from this study provide evidence that even when faced with some of life’s most difficult events, Black middle to older adults were still able to reframe their situation with a positive focus. The ability for Black middle to older aged adults to reframe their tragedies into positivity could provide a basis for the use of Positive Psychological techniques specifically within this population. Additionally, this study provides further evidence that Black people exhibit exceeding resilience.

